# Post-market safety profile and suicide risk signals of vortioxetine: a real-world pharmacovigilance study

**DOI:** 10.3389/fphar.2026.1772885

**Published:** 2026-04-15

**Authors:** Guanguan Qiu, Dan Ye, Xiaomin Xu

**Affiliations:** 1 Shaoxing Second Hospital, Shaoxing, Zhejiang, China; 2 Shaoxing Blood Center, Shaoxing, Zhejiang, China

**Keywords:** adverse event, data mining, FAERS, suicide signals, vortioxetine

## Abstract

**Background:**

Vortioxetine inhibits 5-HT transporter-mediated 5-HT reuptake and simultaneously regulates the activities of various 5-HT receptors to exert an antidepressant effect. It has been proven to be a highly effective antidepressant that can significantly improve depressive symptoms. However, the real-world evidence of vortioxetine-associated suicidal signals is very limited. We explored the vortioxetine-related suicidal signals by mining the US FDA Adverse Event Reporting System (FAERS) database.

**Methods:**

This study used the disproportionality method to systematically evaluate the vortioxetine-related adverse events (AEs). Data were collected from the fourth quarter (Q4) of 2013 to the second quarter (Q2) of 2025 in the FAERS database. The report was analyzed by four signal detection methods, namely, reported odds ratio (ROR), proportional reporting ratio (PRR), Bayesian confidence propagation neural network (BCPNN), and multi-item gamma Poisson shrinker (MGPS). The contingency table was analyzed to compare the proportion of suicide-related reports across different subgroups.

**Results:**

A total of 13,698 vortioxetine-related reports were cleaned from the FAERS database. From these reports, 27 system organ classes (SOCs) and 173 preferred terms (PTs) were identified. The top three SOCs were psychiatric disorders, general disorders and administration site conditions, and gastrointestinal disorders. Significant suicide-related signals were detected for suicidal ideation (ROR = 19.87), suicide attempt (ROR = 7.05), completed suicide (ROR = 4.73), suicidal behavior (ROR = 13.57), and suicide threat (ROR = 15.47). The contingency table analysis revealed that sex (*χ* ^*2*^ = 48.77, *p* < 0.001) and age (*χ* ^*2*^ = 30.17, *p* < 0.001) were significant factors associated with suicide-related reporting. Although the highest proportion of suicide-related reports occurred in the 25- to 64-year age group (12.90%), the ≤24-year age group also contributed a meaningful proportion (8.86%), consistent with the FDA black box warning for antidepressants. No significant association was observed with weight (*p* = 0.64).

**Conclusion:**

This study aligns with regulatory warnings and underscores the importance of clinical vigilance when prescribing vortioxetine. Enhanced monitoring is recommended for all patients, with particular attention given to younger populations during treatment initiation. Due to the inherent limitations of spontaneous reporting systems, these findings should be interpreted as hypothesis-generating signals requiring confirmation through controlled studies.

## 1 Introduction

Psychiatric disorders account for 22.8% of the global burden of diseases (Murray et al., 2015). Depression is the leading cause of this disability burden, which has significantly increased since 1990, primarily due to population growth and aging (Cipriani et al., 2018). Major depressive disorder (MDD) is characterized by persistent feelings of sadness or loss of pleasure in daily activities, along with other cognitive, behavioral, or neurovegetative symptoms. It affects approximately 340 million people globally. MDD places a heavy burden on individuals, families, and society (Yang et al., 2024). The MDD burden is partially contributed by its increased association with suicidal ideation and suicidal behavior, including suicide attempt and suicide death (Chesney et al., 2014). Despite the availability of various drugs and psychotherapeutic interventions, a considerable proportion (approximately one-third) of patients with MDD fail to achieve adequate symptom remission through standard treatments alone (Rush et al., 2006; Trivedi et al., 2006). Therefore, developing novel antidepressants has become a key focus of the current research.

Vortioxetine is a novel antidepressant approved by the U.S. Food and Drug Administration (FDA) in 2013 for the treatment of MDD, jointly developed by Lundbeck and Takeda. Vortioxetine, a 5-HT3, 5-HT7, and 5-HT1D receptor antagonist; a 5-HT1B receptor partial agonist; a 5-HT1A receptor agonist; and a serotonin (5-HT) transporter (SERT) inhibitor (Sanchez et al., 2015), is the first mixed serotonin agonist and antagonist antidepressant. Vortioxetine has been approved for MDD treatment, and clinical trials have demonstrated its efficacy in improving depressive symptoms, cognitive function, and quality of life, with a relatively mild side-effect profile (Verrienti et al., 2025; Xue et al., 2025; Ibáñez et al., 2024). However, like all antidepressants, the potential for treatment-emergent suicidality warrants continued scrutiny. A pivotal *post hoc* pooled analysis of ten randomized placebo-controlled trials and three open-label extension studies demonstrated that vortioxetine was not associated with increased suicidal ideation or behavior in adult MDD patients, with no completed suicides across all studies (Mahableshwarkar et al., 2020). Although these clinical trial findings are reassuring, they are inherently constrained by stringent selection criteria, limited statistical power to detect rare events, and restricted follow-up durations, which may not fully reflect the spectrum of suicide-related events encountered in real-world clinical practice. It is well established that spontaneous reporting databases such as FDA Adverse Event Reporting System (FAERS) are uniquely positioned to capture rare but clinically significant adverse events (AEs) that may not emerge during the pre-marketing trial phase.

The FAERS database supports the FDA’s post-marketing safety surveillance program for all marketed drug and therapeutic biologic products. It helps the FDA promptly identify potential safety signals by collecting AE reports submitted by healthcare professionals and consumers (He et al., 2025; Liu et al., 2025). Due to its huge volume of data and public accessibility, the FAERS database has become an important tool for researchers to assess the safety of drugs (Zhao et al., 2024). Researchers utilize the FAERS database to collect data pertaining to a range of medications and diseases to identify AEs that require special attention (Yuan et al., 2025). However, real-world safety data for vortioxetine with suicide-related signals remain limited. Therefore, we employed a combination of four disproportionality analysis methods to investigate vortioxetine and suicide-related AEs within the FAERS database. The findings aim to provide critical evidence for guiding the safe and rational clinical application of vortioxetine.

## 2 Methods

### 2.1 Study design and data source

AE reports in ASCII format from the fourth quarter (Q4) of 2013 to the second quarter (Q2) of 2025 were retrieved from the FAERS database. Tables containing report sources, demographic information (DEMO), drug information (DRUG), AEs, and patient outcomes were linked and integrated using the primary ID. Although the FAERS database is managed primarily by the United States, it incorporates AE reports from worldwide sources, making this comprehensive open database particularly suitable for research on adverse drug reactions. As the FAERS database is publicly accessible and all patient records are anonymized, neither informed consent nor ethical approval was required for this study.

### 2.2 Data extraction and analysis

**FIGURE 1:**
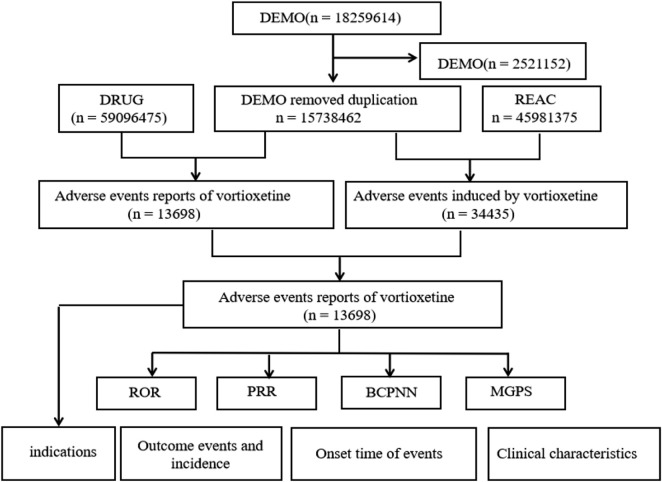
Flow diagram for the selection of AEs associated with vortioxetine from FAERS database.

Data cleaning was performed following FDA-recommended procedures for duplicate removal. Specifically, the Primary ID, Case ID, and FDA_DT fields were extracted from the DEMO table. Records were sorted by Case ID, FDA_DT, and Primary ID. For reports sharing the same Case ID, those with the latest FDA_DT value were retained. If the Case ID and FDA_DT were identical, the record with the highest Primary ID was selected. The reports of “Lu AA21004,” “Brintellix,” “Vortioxetine Hydrobromide,” “Vortioxetine,” “Vortioxetine hbr,” and “1-(2-(2,4-Dimethylphenylsulfanyl)phenyl)piperazine” as the primary suspect (PS) drugs were screened for analysis. Figure 1 presents the process of data extraction and mining. The system organ classes (SOCs) and preferred terms (PT) were defined according to the Regulatory Activities Medical Dictionary of 28.0 (MedDRA 28.0). The drug-related suicide-associated PTs such as suicidal ideation, suicidal behavior, depression suicidal, suicide attempts, and completed suicide were informed by prior studies (Liu et al., 2020).

### 2.3 Statistical analysis

Data were analyzed using R software (version 4.5.1) and Microsoft Excel software (version 2017, Microsoft Corporation). The data reported odds ratio (ROR) (Rothman et al., 2004), proportional reporting ratio (PRR) (Evans et al., 2001), multi-item gamma Poisson shrinker (MGPS) (DuMouchel, 1999), and Bayesian confidence propagation neural network (BCPNN) (Bate et al., 1998) to explore the AE signals of vortioxetine. A two-by-two contingency table and detailed formulae for disproportionality analysis are shown in Supplementary Tables S1, S2. The positive signal required the concurrent satisfaction of all four algorithmic criteria.

SPSS 27 software was applied for statistical analysis. Between-group differences in the proportion of suicide-related reports were evaluated using Pearson’s chi-square test or Fisher’s exact test. A *p*-value of less than 0.05 was regarded as statistically significant.

## 3 Results

### 3.1 Characteristics of vortioxetine-related AE reports

After data cleaning, a total of 13,698 (number of reports) vortioxetine-related AE reports were recorded in the FAERS database from 2013 Q4 to 2025 Q2. The characteristics of the reports for vortioxetine are shown in Table 1. The reports showed a higher prevalence of female individuals than that of male individuals (60.83% vs. 26.57%), while 12.61% of reports lacked gender information. Patients aged 18–64 years accounted for 34.11% of reports, those aged ≥65 for 8.23%, and those under 18 for 7.96%. Age information was missing in 49.69% of reports, which limited further investigation into the influence of age on AEs. Patients weighing 50–100 kg accounted for 15.83% of reports, but weight data were missing for 80.14%. The United States accounted for 77.05% of the AE reports, and most AE reports were submitted by consumers (49.90%). Regarding the reported outcomes, hospitalization, death, life-threatening, and disability events were reported in 11.17%, 3.10%, 1.59%, and 1.47%, respectively. The highest number of reports was reported in 2017 (14.38%). Subsequently, the number of reports gradually decreased.

**TABLE 1:** Characteristics of all reports treated with vortioxetine.

Characteristic	Number of events	Proportion (%)
Number of reports	13,698	​
Gender		
Female	8,332	60.83%
Male	3,639	26.57%
Missing	1,727	12.61%
Age (years)		
<18	1,091	7.96%
18–64	4,672	34.11%
65–85	1,040	7.59%
>85	88	0.64%
Missing	6,807	49.69%
Weight (kg)		
<50	171	1.25%
50–100	2,169	15.83%
>100	380	2.77%
Missing	10,978	80.14%
Top five reported countries		
United States	10,554	77.05%
Japan	586	4.28%
France	402	2.93%
Canada	274	2.00%
Great Britain	206	1.50%
Reporter		
Consumer	6,835	49.90%
Physician	3,569	26.05%
Healthcare professional	960	7.01%
Pharmacist	352	2.57%
Missing	1,982	14.47%
Outcome		
Congenital anomaly	17	0.12%
Death	425	3.10%
Disability	201	1.47%
Hospitalization	1,530	11.17%
Life-threatening	218	1.59%
Other	11,307	82.54%
Reporting year		
2014	1,043	7.61%
2015	1,433	10.46%
2016	1,406	10.26%
2017	1,970	14.38%
2018	1,573	11.48%
2019	1,495	10.91%
2020	1,113	8.13%
2021	999	7.29%
2022	845	6.17%
2023	735	5.37%
2024	733	5.35%
2025	353	2.58%

### 3.2 Cumulative incidence of vortioxetine-related AEs and the Weibull distribution

**FIGURE 2:**
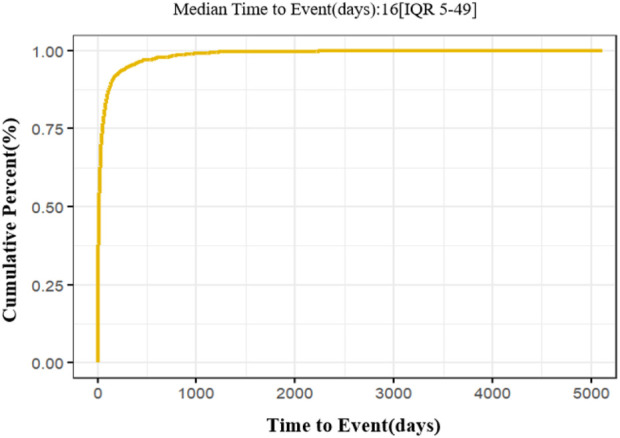
Cumulative incidence of vortioxetine-related AEs.

Figure 2 presents the cumulative occurrence of AEs associated with vortioxetine over time. The median occurrence time of AEs was 16 days. It indicated that vortioxetine-related AEs were most common within the first month of therapy. The Weibull distribution was used to assess the trend of AE occurrence over time. The shape parameter (*β*) was used to characterize the dynamics of the failure rate over time. A *β*-value <1, with its 95% confidence interval (CI) also remaining below 1, is indicative of an early failure pattern. In this pattern, the frequency of AEs is initially elevated but declines over time. If *β* = 1 and its 95% CI includes 1, a constant failure rate, implying a stable risk of AEs throughout the treatment period, is suggested. Conversely, a *β*-value >1, with its 95% CI excluding 1, reflects a wear-out failure pattern, where the risk of AEs increases over time, indicating a significant trend of increasing risk with an extended treatment duration. Weibull distribution analysis indicated an early failure model, with a shape parameter (*β*) of 0.58 (95% CI: 0.56–0.60). The result is shown in Table 2. The risk of the event is highest in the early phase of treatment and decreases significantly over time. It was found that AEs manifest most frequently in the initial stages of therapy. Therefore, clinicians should closely monitor patients during the initial stages of vortioxetine treatment to ensure drug safety.

**TABLE 2:** Cumulative incidence of vortioxetine-related AEs and the Weibull distribution.

Median time to event (days)	Weibull distribution	
Median (*IQR*)	*β* (95% CI)	Type
16 (5,49)	0.58 (95% CI: 0.56–0.60)	Early failure

*IQR*, interquartile range; *CI*, confidence interval.

### 3.3 Analysis of SOCs and PTs related to vortioxetine

The SOC level of vortioxetine-related AEs is shown in Table 3, in which 27 organ systems were reported. The top three SOCs ranked by the number of reports were psychiatric disorders (n = 7,959), general disorders and administration site conditions (n = 5,057), and gastrointestinal disorders (n = 5,010). Notably, psychiatric disorders were the only SOCs that met the criteria of the four algorithms simultaneously.

**TABLE 3:** Distribution of AEs of target drugs in different SOCs.

System organ classification	n	ROR (95% Cl)	PRR (chi-square)	EBGM (EBGM_05_)	IC (IC_025_)
Psychiatric disorders	7,959	5.41 (5.28–5.55)	4.39 (21,939.67)	4.38 (4.27)	2.13 (2.09)
General disorders and administration site conditions	5,057	0.79 (0.77–0.81)	0.82 (242.94)	0.82 (0.8)	−0.29 (−0.33)
Gastrointestinal disorders	5,010	1.87 (1.82–1.93)	1.74 (1732.52)	1.74 (1.69)	0.80 (0.76)
Nervous system disorders	4,416	1.72 (1.66–1.77)	1.63 (1153.81)	1.63 (1.57)	0.70 (0.65)
Injury, poisoning, and procedural complications	2,665	0.64 (0.61–0.66)	0.67 (501.67)	0.67 (0.64)	−0.58 (−0.64)
Skin and subcutaneous tissue disorders	2,036	1.06 (1.01–1.1)	1.05 (5.7)	1.05 (1.01)	0.07 (0.01)
Investigations	1,376	0.68 (0.65–0.72)	0.69 (197.04)	0.69 (0.66)	−0.53 (−0.61)
Musculoskeletal and connective tissue disorders	727	0.39 (0.37–0.42)	0.41 (663.65)	0.41 (0.38)	−1.30 (−1.4)
Eye disorders	658	0.97 (0.9–1.05)	0.97 (0.66)	0.97 (0.9)	−0.05 (−0.16)
Metabolism and nutrition disorders	606	0.85 (0.78–0.92)	0.85 (15.74)	0.85 (0.79)	−0.23 (−0.35)
Surgical and medical procedures	511	1.04 (0.95–1.13)	1.04 (0.64)	1.04 (0.95)	0.05 (−0.08)
Reproductive system and breast disorders	481	1.74 (1.59–1.9)	1.73 (148.47)	1.73 (1.58)	0.79 (0.65)
Respiratory, thoracic, and mediastinal disorders	466	0.28 (0.26–0.31)	0.29 (833.53)	0.29 (0.27)	−1.77 (−1.9)
Cardiac disorders	462	0.6 (0.55–0.66)	0.6 (122.08)	0.6 (0.55)	−0.73 (−0.86)
Infections and infestations	320	0.16 (0.15–0.18)	0.17 (1349.23)	0.17 (0.15)	−2.54 (−2.7)
Vascular disorders	309	0.45 (0.4–0.5)	0.45 (208.99)	0.45 (0.4)	−1.14 (−1.31)
Renal and urinary disorders	258	0.40 (0.35–0.45)	0.4 (236.28)	0.40 (0.35)	−1.32 (−1.5)
Ear and labyrinth disorders	193	1.30 (1.13–1.5)	1.30 (13.13)	1.30 (1.13)	0.37 (0.16)
Social circumstances	184	1.19 (1.03–1.38)	1.19 (5.56)	1.19 (1.03)	0.25 (0.04)
Immune system disorders	144	0.35 (0.3–0.41)	0.35 (170.96)	0.35 (0.3)	−1.49 (−1.73)
Neoplasms benign, malignant, and unspecified (including cysts and polyps)	118	0.12 (0.1–0.14)	0.12 (756.56)	0.12 (0.1)	−3.02 (−3.27)
Hepatobiliary disorders	114	0.39 (0.33–0.47)	0.39 (106.91)	0.39 (0.33)	−1.34 (−1.6)
Pregnancy, puerperium, and perinatal conditions	114	0.86 (0.71–1.03)	0.86 (2.7)	0.86 (0.71)	−0.22 (−0.49)
Blood and lymphatic system disorders	99	0.17 (0.14–0.21)	0.18 (391.05)	0.18 (0.14)	−2.51 (−2.79)
Endocrine disorders	87	0.97 (0.79–1.2)	0.97 (0.08)	0.97 (0.79)	−0.04 (−0.35)
Congenital, familial, and genetic disorders	33	0.35 (0.25–0.5)	0.35 (39.26)	0.35 (0.25)	−1.5 (−1.97)
Product issues	32	0.05 (0.04–0.07)	0.05 (571.81)	0.05 (0.04)	−4.28 (−4.74)

*ROR*, reporting odds ratio; *PRR*, proportional reporting ratio; *IC*, information component; *EBGM*, empirical Bayesian geometric mean.

Vortioxetine identified 2,090 signals at the PT levels, with 173 significantly disproportionate PTs satisfying all four algorithms simultaneously. The top 20 positive AEs reports for vortioxetine at the PT level are shown in Table 4. The top five signals with the highest numbers of reports were nausea (n = 2,040, ROR = 5.07, PRR = 4.83, IC = 2.27, and EBGM = 4.82), suicidal ideation (n = 847, ROR = 19.87, PRR = 19.41, IC = 4.26, and EBGM = 19.15), anxiety (n = 783, ROR = 5.28, PRR = 5.18, IC = 2.37, and EBGM = 5.16), vomiting (n = 773, ROR = 3.2, PRR = 3.15, IC = 1.65, and EBGM = 3.15), and pruritus (n = 762, ROR = 3.57, PRR = 3.51, IC = 1.81, and EBGM = 3.5). FAERS contains AE reports that are not necessarily drug-related; it also contains several non-vortioxetine-induced AE signals, such as drug name confusion, no adverse event, and product prescribing error.

**TABLE 4:** The top 20 positive signal strength of AE reports for vortioxetine at the PT levels in the FAERS database.

SOC	PT	n	ROR (95% Cl)	PRR (chi-square)	EBGM (EBGM_**05**_)	IC (IC_**025**_)
Gastrointestinal disorders	Nausea	2,040	5.07 (4.85–5.3)	4.83 (6248.88)	4.82 (4.6)	2.27 (2.2)
Psychiatric disorders	Suicidal ideation	847	19.87 (18.55–21.29)	19.41 (14,595.96)	19.15 (17.87)	4.26 (4.13)
Psychiatric disorders	Anxiety	783	5.28 (4.91–5.66)	5.18 (2641.48)	5.16 (4.81)	2.37 (2.26)
Gastrointestinal disorders	Vomiting	773	3.20 (2.98–3.44)	3.15 (1141.57)	3.15 (2.93)	1.65 (1.55)
Skin and subcutaneous tissue disorders	Pruritus	762	3.57 (3.32–3.83)	3.51 (1372)	3.50 (3.26)	1.81 (1.7)
General disorders and administration site conditions	Feeling abnormal	609	4.63 (4.28–5.02)	4.57 (1699.36)	4.56 (4.21)	2.19 (2.06)
Psychiatric disorders	Insomnia	584	4.19 (3.86–4.55)	4.13 (1389.4)	4.13 (3.8)	2.04 (1.92)
General disorders and administration site conditions	No adverse event	518	4.82 (4.42–5.26)	4.76 (1538.98)	4.75 (4.35)	2.25 (2.11)
General disorders and administration site conditions	Asthenia	490	2.43 (2.23–2.66)	2.41 (407.06)	2.41 (2.2)	1.27 (1.13)
Investigations	Weight increased	469	3.98 (3.63–4.36)	3.94 (1028.65)	3.93 (3.59)	1.97 (1.83)
Psychiatric disorders	Irritability	437	14.54 (13.22–15.98)	14.36 (5380.43)	14.22 (12.93)	3.83 (3.65)
Nervous system disorders	Disturbance in attention	409	14.57 (13.21–16.07)	14.4 (5051.74)	14.26 (12.93)	3.83 (3.64)
Psychiatric disorders	Anger	400	24.74 (22.4–27.33)	24.47 (8845.32)	24.04 (21.77)	4.59 (4.36)
Psychiatric disorders	Depression	374	3.33 (3.01–3.69)	3.31 (601.76)	3.3 (2.98)	1.72 (1.56)
Gastrointestinal disorders	Constipation	314	2.62 (2.34–2.93)	2.61 (311.01)	2.6 (2.33)	1.38 (1.21)
Psychiatric disorders	Apathy	292	38.99 (34.69–43.83)	38.67 (10,416.24)	37.61 (33.46)	5.23 (4.89)
Psychiatric disorders	Agitation	263	7.55 (6.69–8.53)	7.50 (1475.24)	7.47 (6.61)	2.90 (2.69)
Skin and subcutaneous tissue disorders	Hyperhidrosis	239	3.60 (3.17–4.09)	3.59 (445.27)	3.58 (3.15)	1.84 (1.64)
Nervous system disorders	Hypersomnia	223	14.54 (12.74–16.6)	14.45 (2763.55)	14.31 (12.53)	3.84 (3.56)
Psychiatric disorders	Mood swings	220	14.09 (12.33–16.09)	14 (2629.74)	13.87 (12.14)	3.79 (3.52)

### 3.4 Characteristics of vortioxetine-related suicide reports

A total of 1,273 suicide-related reports associated with vortioxetine were identified in the FAERS database during the study period. Table 5 presents the clinical features of vortioxetine-related suicide reports. The reports showed a higher proportion of female individuals than male individuals (55.62% vs. 36.06%). The contingency table analysis revealed that sex was a significant factor associated with suicide-related reporting (*χ* ^*2*^ = 48.77, *p* < 0.001) (Table 6). Patients aged 18–64 years accounted for the highest proportion (44.78%), but age information was missing in 40.61% of the reports. The contingency table analysis suggested that age is significantly associated with suicide-related reporting (*χ* ^*2*^ = 30.17, *p* < 0.001). The proportion of the 50–100 kg group was 15.87%, but 79.89% of the reports lacked weight data. No statistically significant association was observed between the weight category and suicide-related reporting (*χ* ^*2*^ = 0.88, *p* = 0.64). Regarding the reported outcomes, death, hospitalization, life-threatening, and disability events were reported in 194 (15.24%), 172 (13.51%), 105 (8.25%), and 12 (0.94%) reports, respectively.

**TABLE 5:** Clinical characteristics of vortioxetine-related suicide reports.

Characteristic	Vortioxetine-related suicide reports (N = 1273)	Proportion (%)
Gender		
Female	708	55.62%
Male	459	36.06%
Missing	106	8.33%
Age (years)		
<18	109	8.56%
18–64	570	44.78%
65–85	75	5.89%
>85	2	0.16%
Missing	517	40.61%
Weight (kg)		
<50	14	1.10%
50–100	202	15.87%
>100	40	3.14%
Missing	1,017	79.89%
Outcome		
Death	194	15.24%
Life-threatening	105	8.25%
Hospitalization	172	13.51%
Disability	12	0.94%
Other serious important medical events	790	62.06%

**TABLE 6:** Patient demographics (sex, age, and weight) in vortioxetine-associated suicide reports.

Characteristic	Non-suicidal-related reports	Suicide-related reports	Total reports	χ^2^	*P*
Gender					
Female	7,624	708	8,332	48.77	0.00
Male	3,180	459	3,639		
Age (years)					
<18	982	109	1,091	30.17	0.00
18–64	4,102	570	4,672		
65–85	965	75	1,040	​	​
>85	86	2	88	​	​
Weight (kg)					
<50	157	14	171	0.88	0.64
50–100	1,967	202	2,169		
>100	340	40	380		

### 3.5 Descriptive analysis of suicide-related PTs

Vortioxetine-related suicide reports at the PT levels were ranked, as shown in Table 7. The top three signals with the highest numbers of reports were suicidal ideation (n = 847, ROR = 19.87, PRR = 19.41, IC = 4.26, and EBGM = 19.15), suicide attempt (n = 204, ROR = 7.05, PRR = 7.02, IC = 2.8, and EBGM = 6.98), and completed suicide (n = 185, ROR = 4.73, PRR = 4.71, IC = 2.23, and EBGM = 4.69). PTs of suicidal ideation, suicide attempt, completed suicide, suicidal behavior, and suicide threat satisfied all four algorithms simultaneously.

**TABLE 7:** Vortioxetine-related suicide reports at the PT levels in the FAERS database.

PT	n	ROR (95% Cl)	PRR (chi-square)	EBGM (EBGM_05_)	IC (IC_025_)
Suicidal ideation	847	19.87 (18.55–21.29)	19.41 (14,595.96)	19.15 (17.87)	4.26 (4.13)
Suicide attempt	204	7.05 (6.14–8.09)	7.02 (1,047.54)	6.98 (6.08)	2.80 (2.56)
Completed suicide	185	4.73 (4.09–5.46)	4.71 (538.6)	4.69 (4.06)	2.23 (1.99)
Suicidal behavior	28	13.57 (9.35–19.69)	13.56 (322.38)	13.43 (9.25)	3.75 (2.69)
Suicide threat	4	15.47 (5.77–41.46)	15.47 (53.52)	15.3 (5.71)	3.94 (0.69)
Depression suicidal	4	2.27 (0.85–6.06)	2.27 (2.84)	2.27 (0.85)	1.18 (−0.44)
Columbia suicide severity rating scale abnormal	1	1,334.35 (83.46–21,334.42)	1,334.31 (666.16)	667.65 (41.76)	9.38 (−1.64)

## 4 Discussion

Traditional selective serotonin reuptake inhibitors (SSRIs) or serotonin–norepinephrine reuptake inhibitors (SNRIs) exert antidepressant effects primarily through serotonin and/or norepinephrine reuptake blockade. The resultant 2- to 4-week delay in the therapeutic onset is frequently accompanied by transient adverse effects (including anxiety, agitation, and sleep disturbance),which may increase the suicidality risk during early treatment (Jick et al., 2004). Vortioxetine is a novel multimodal antidepressant that has emerged as a promising option for the management of MDD (Zhang et al., 2022). Vortioxetine exerts its antidepressant effects through a combination of serotonin reuptake inhibition, receptor modulation, and neuroplasticity enhancement (Stahl, 2015; Kugathasan et al., 2017). The pharmacological profile of vortioxetine involves serotonin transporter (SERT) inhibition combined with receptor modulation: 5-HT1A agonism, 5-HT1B partial agonism, and antagonism at 5-HT3, 5-HT7, and 5-HT1D receptors (Stahl, 2015). This pharmacology may confer more rapid anxiolytic and mood-stabilizing effects, thereby potentially mitigating the early activation syndrome (characterized by anxiety, agitation, and restlessness) (Stahl, 2015). Preclinical studies suggest that 5-HT3 receptor antagonism may reduce anxiety-related behaviors more rapidly than reuptake inhibition alone (Mork et al., 2013). The safety data of vortioxetine primarily originate from clinical trial reports. However, although the safety profile of vortioxetine is well characterized from clinical trials, real-world evidence about AEs, particularly suicidality, remains limited. We retrospectively reviewed the FAERS database to identify reports of suicide-related events associated with vortioxetine. The objective is to provide evidence-based data that can inform updates to prescribing information and promote the safe and rational use of this drug in clinical practice.

In this study, female subjects reported more AEs than male subjects, which is consistent with the epidemiological investigation of depression by sex (Salk et al., 2017). Many studies focused on biological systems involved in MDD pathophysiology, including the stress response, inflammatory and immune systems, monoaminergic, neurotrophic, gamma-aminobutyric acid and glutamatergic systems, the oxytocin system, the endocrine system, and sex-specific hormone effects, which contribute to this sex disparity (Silva et al., 2023). Most AEs were reported in the 18- to 64-year age group, which may correlate with the increased incidence of depression observed (Thapar et al., 2022). Additionally, the United States reported the greatest number of AEs, followed by Japan and France. This difference was mainly attributed to three factors: first, the United States had a larger population base, and vortioxetine was first marketed in the United States; second, its adverse drug reaction monitoring system is more comprehensive; third, there are regional differences in antidepressant prescribing patterns (Zhou et al., 2025).

At the SOC level, the top three significant signals were psychiatric disorders, gastrointestinal system, and nervous system, consistent with other reports. Many clinical studies reported that post-treatment adverse effects frequently occur in the psychiatric, neurological, and gastrointestinal systems (Sanchez et al., 2015; Findling et al., 2022). This study analyzed the top 100 positive signal strength of AE reports for vortioxetine at the PT levels. The top 10 serious AEs were nausea, suicidal ideation, anxiety, vomiting, pruritus, feeling abnormal, insomnia, asthenia, weight increase, and irritability. The AEs listed in the FDA labeling corresponded to the clinical study data (Meeker et al., 2015).

Psychiatric disorders were the most frequently reported vortioxetine-related AEs. We identified a total of 1,273 reports of suicidal behavior associated with vortioxetine in the FAERS database from 2013 Q4 to 2025 Q2. Suicide-related AE reports showed a significantly higher proportion of female individuals (55.62%) than male individuals (36.06%), and contingency table analysis confirmed sex as a significant factor (*χ* ^*2*^ = 48.77, *P* < 0.001). The gender differences are consistent with reports of previous epidemiological investigations of suicide (DelBello et al., 2025). Age was significantly associated with suicide-related reporting (*χ* ^*2*^ = 30.17, *P* < 0.001). Although the highest proportion of suicide-related reports occurred in the 25-to 64-year age group (12.90%), the ≤24-year group also contributed a meaningful proportion (8.86%) (Supplementary Table S4). This finding is of critical importance as it aligns with the FDA’s black box warning for all antidepressants: a warning that specifically cautions about an increased signal for suicidal thinking and behavior in children, adolescents, and young adults (Mahableshwarkar et al., 2020). In the long-term extension studies, children and adolescents treated with vortioxetine showed good tolerability in patients with MDD, and no increased risk was observed (DelBello et al., 2025). In this study, it is recommended that children, adolescents, and young adults treated with vortioxetine for any indication undergo appropriate monitoring and close observation for clinical worsening, suicidality, and unusual changes in behavior. In addition, no significant association was found with weight (*P* = 0.64)*,* suggesting that this factor may not be a key differentiator in suicide-related AEs for vortioxetine.

Vortioxetine-related suicide reports included suicidal ideation, suicide attempt, completed suicide, suicidal behavior, suicide threat, suicidal depression, and Columbia suicide severity rating scale abnormal. Among the seven categories of suicide reports, the signal with the highest number of reports was suicidal ideation. This result was in line with that of a study based on the VigiBase database (Ekhart et al., 2022). The PT of the Columbia suicide severity rating scale abnormal was derived from only one report (n = 1), resulting in an extremely wide confidence interval (ROR = 1,334.35, 95% CI: 83.46–21,334.42) and IC_025_ that crossed zero (IC = 9.38, IC_025_ = −1.64). This finding illustrates a fundamental principle of pharmacovigilance signal detection: the precision and stability of disproportionality estimates depended on the underlying number of reports (Sardella and Lungu, 2019; Noguchi and Yoshimura, 2024).

The Weibull distribution analysis demonstrated an early failure model (*β* = 0.58, 95% CI: 0.56–0.60). It indicated that AE reports (including suicide-related events) peaked in the initial phase of treatment (median onset time: 16 days) and decreased over time. This finding supported current clinical recommendations for close patient monitoring, particularly for worsening depression and suicidality during the first few months of therapy.

MDD has been identified as a risk factor for suicidal behavior. Antidepressants play a crucial role in MDD. Vortioxetine has demonstrated efficacy in improving clinical outcomes for patients with MDD. This is supported by evidence from 11 placebo-controlled trials, as well as active-controlled and relapse-prevention studies (Sanchez et al., 2015). Vortioxetine’s unique psychopharmacological profile, which distinguishes it from other antidepressants, is believed to underpin its therapeutic benefits in MDD. However, the debate over the potential association between antidepressants and an increased risk of suicidal ideation during treatment continues. Indeed, suicidality is a core symptom of MDD, and meta-analyses of completed suicides demonstrated that the likelihood of dying by suicide owing to mood disorders is 8.62 times higher in MDD patients compared to the general population (Arnone et al., 2024). Consequently, any analysis of suicide-related AEs associated with an antidepressant must contend with the fundamental challenge of disentangling drug-attributable events from disease-inherent manifestations.

This confounding by indication represents an inherent limitation of pharmacovigilance studies utilizing spontaneous reporting databases such as FAERS, which lack detailed clinical information regarding disease severity, duration, and psychiatric comorbidities (Lee and Chang, 2022; McIntyre et al., 2025). Therefore, our findings should be interpreted as hypothesis-generating signals that warrant clinical vigilance, rather than as evidence of a causal relationship between vortioxetine and suicidality (Fusaroli et al., 2026; Xing et al., 2025). Future research utilizing linked databases with comprehensive clinical phenotyping, or meta-analyses of randomized controlled trial data, would be better positioned to disentangle drug effects from disease effects.

This study has several limitations. First, the FAERS database is a spontaneous reporting system. The reports, which can be submitted by consumers and healthcare professionals (such as physicians, nurses, and pharmacists), frequently contain incomplete data, which precludes the calculation of true incidence rates. Notably, a substantial proportion of reports had missing age data: 49.69% of vortioxetine-related reports and 40.61% of suicide-related reports. This high rate of incomplete demographic reporting represents a significant methodological constraint, potentially introducing selection bias and reducing statistical precision in age-stratified analyses. Importantly, the missing data were not randomly distributed across age groups, which could disproportionately affect the reliability of estimates for certain populations, particularly the ≤24-year and 25- to 64-year age groups. The higher proportion of reports in the 25- to 64-year age group may reflect greater vortioxetine utilization in this population rather than an intrinsically higher risk. Without denominator data on prescribing volume by age group, we cannot calculate true incidence rates or determine whether the observed patterns reflect differential prescribing practices, differential reporting behaviors, or differential underlying risk. Furthermore, the reports are predominantly from the United States. The lack of comprehensiveness has the potential to compromise data integrity and introduce bias into subsequent analyses. Second, the FAERS database has inherent limitations for analytical sensitivity, including the absence of key clinical details such as comprehensive patient profiles and precise treatment timelines. This limitation critically impedes our ability to distinguish vortioxetine-related suicide from disease-inherent manifestations. Therefore, causality cannot be established by disproportionality analysis alone. Individual case review and confirmation in other data sources are essential before a signal is considered a confirmed risk. Finally, some AEs involved multiple suspect drugs, including SSRIs, atypical antipsychotics, and atypical antidepressants; therefore, it cannot be determined whether these AEs were associated with vortioxetine alone.

## 5 Conclusion

This study identified significant disproportionate reporting of suicide-related events associated with vortioxetine in the FAERS database, with notable age-related heterogeneity (χ^2^ = 45.02, p < 0.001). Although the highest proportion of reports was observed in the 25- to 64-year age group (12.90%), elevated signals were also detected in the ≤24-year age group (8.86%), which is consistent with FDA regulatory warnings for administration of antidepressants in young populations. These findings highlight the need for clinical vigilance and a careful risk–benefit assessment when prescribing vortioxetine, with particular attention to younger patients. Clinicians should remain aware of these signals while recognizing their limitations. Prescribing decisions should be based on a careful assessment of individual patient characteristics, in alignment with clinical guidelines and regulatory recommendations.

## Data Availability

The original contributions presented in the study are included in the article/Supplementary Material; further inquiries can be directed to the corresponding author.
